# Outcomes from a pilot psychological therapies service for UK military veterans

**DOI:** 10.1002/nop2.57

**Published:** 2016-06-28

**Authors:** Paul Clarkson, Clarissa M. Giebel, David Challis, Paul Duthie, Alan Barrett, Helen Lambert

**Affiliations:** ^1^ Personal Social Services Research Unit (PSSRU)University of ManchesterManchesterUK; ^2^ Pennine Care NHS Foundation TrustLancashireUK; ^3^ Military Veterans’ IAPT Service (North West)Pennine Care NHS Foundation TrustBuryUK

**Keywords:** adult nursing, audit, cognitive therapy, depression, mental health

## Abstract

**Aim:**

To evaluate the outcomes of participants attending a psychological therapies service for military veterans.

**Background:**

The UK Military Veterans’ Improving Access to Psychological Therapies Service (North West) (MV IAPT) provided a clinical psychological therapies service for military veterans. Outcomes of depression, anxiety and social adjustment were assessed after treatment in the service's pilot phase.

**Design:**

An observational, prospective cohort study examined changes in depression, anxiety and social adjustment during receipt of the service.

**Methods:**

Changes in depression (PHQ‐9), anxiety (GAD‐7) and social adjustment (WSAS) were examined in 952 veterans referred over 20 months from September 2011. Data were collected using the IAPT clinical information system plus additional fields. Changes for patients who completed treatment, remained in treatment and dropped out were compared.

**Results:**

Seven hundred and seven veterans received an initial assessment, from which 505 received two or more appointments. Of these, 156 completed treatments, 179 remained in treatment and 170 dropped out. The majority of veterans had been operationally deployed and were similar in risk characteristics to those in other military cohort studies. There were highly significant improvements on all measures (*p*<.01), with completers improving more and having higher rates of recovery from depression and anxiety than those remaining and drop outs. Recovery rates compared favourably with evaluations of general IAPT services and also exceeded reported natural recovery rates.

## 1 Introduction

Each year, a proportion of personnel leave military service, which may place strain on their mental and physical well‐being as well as their successful reintegration into society. Returning from war zones into civilian life can precipitate stress or further exacerbate existing mental health problems. However, military veterans represent a vulnerable and marginalised group, relatively ill‐served by traditional forms of health and social care (Woodhead et al., 2011). To help address these problems, specific services for veterans are available in different countries, depending on circumstances and statutory and legal positions in each jurisdiction. In the UK, there are now obligations to meet the specific health and social needs of veterans with targeted approaches, which recognize their unique circumstances and ways of accessing services (Ministry of Defence 2011).

Outflow from the military has remained comparatively constant over recent years, with approximately 11% of UK military personnel becoming civilians each year (Royal British Legion 2006). While veterans of the UK Armed Forces do not necessarily suffer psychological disorder, a significant minority do (Hotopf et al., 2006; Iversen, Dyson, et al., 2005; Iversen, Nikolaou, et al., 2005). Those that do are more than likely not to have their mental health problems identified and treated. Moreover, certain groups of veterans appear to fair particularly badly in terms of symptom levels and eventual outcomes. These include those who left the services early, those with physical disability, reservists and those misusing drugs or alcohol (Browne et al., 2007; Buckman et al., 2013; Iversen, Nikolaou, et al., 2005; Lee, Gabriel, Bolton, Bale, & Jackson, 2002). These groups may require specifically tailored interventions to help resolve problems such as symptoms of depression and anxiety and high levels of social maladjustment, including difficulties in seeking help with housing, finance and employment. Recommendations made by Murrison (2010) to tackle these problems include combating the consequences of stigma, in terms of ensuring that interventions are acceptable to a population accustomed to viewing itself as mentally and physically robust.

### 1.1 Background

In this respect, there have been efforts towards designing specific clinical and social services for UK veterans and these are developing, but have been little evaluated. These services include the UK Medical Assessment Programme (now known as the Veterans and Reserves Mental Health Programme) (Palmer, 2012), directed towards assessing the specific needs of veterans and the Community Housing and Therapy initiative (Gale, Saftis, Vidana, & Sanchez, 2008), offering psychological and social support to homeless veterans. Interventions whose outcomes have been evaluated include the Reserves Mental Health Programme (RMHP) (Jones et al., 2011) and the Community Mental Health Pilots for veterans (Dent‐Brown et al., 2010), both of which have shown good outcomes, for example, improved mood and social adjustment, although the numbers included in analyses were small. It appears, from international systematic reviews of psychosocial interventions for veterans (Kitchiner, Roberts, Wilcox, & Bisson, 2012), that solid evidence of their efficacy is lacking and this has resulted in debate as to the most useful models for veteran‐specific mental health services in the UK (Macmanus & Wessely, 2013). There is no denying a recent political commitment and obligation to provide mental health care that serves the specific needs of veterans (Forster, 2012) but robust evidence of different ways of providing this is still required.

As part of this recent commitment to developing veteran‐specific mental health services in the UK, several regional NHS services have been funded. One model of delivery employs the framework adopted by the Improving Access to Psychological Therapy (IAPT) services (Clark, 2011) for treating depression and anxiety disorders, but configures this specifically for veterans. One such service in this vein is the Military Veterans’ Improving Access to Psychological Therapies Service (North West) (MV IAPT), a specialist IAPT service providing a range of clinical psychological therapies specifically for military veterans, following a ‘stepped care’ model, focusing on the specific needs of patients who struggled to engage with local IAPT services. The service was funded through previous Strategic Health Authority monies for two years (1 April 2011 – 31 March 2013) as a primary care mental health service providing non‐urgent care. The MVIAPT service aimed to provide psychological therapies to veterans who were unwilling or unable to access their local primary care/IAPT services. The criteria for inclusion in the service were:

- Being a military veteran or a family member presenting with mild to moderate mental health difficulties that would benefit from non‐urgent psychologically informed input.
- A military veteran was defined as: (i) a person who has served in any of the British Armed Forces for a day or more; (ii) a person who has served in the British Territory Army, British Royal Navy or British RAF reserves; or (iii) a person who has served in the British Merchant Navy who has experienced active tours of duty.
- The service was unable to see anyone currently serving in the Armed Forces.

The majority of referrals to the service were self‐referrals and referrals from third sector organisations working with veterans. However, referrals were also accepted from NHS mental health services, criminal justice services, employers and others. Most referrals received an initial telephone triage to assess suitability for the service, provide a basic risk assessment and identify type of intervention required. Those deemed not suitable for the service were referred to more appropriate services – usually secondary care mental health services, local primary care services or non‐clinical support. Substance misuse and serious forensic history were not barriers to acceptance by the service. Where necessary patients were supported to access addiction services and able to access therapy alongside addiction services as soon as appropriate.

Patients retained in the service were treated by therapists, both High Intensity Therapists (HITs), predominantly Registered Mental Health Nurses and Psychological Wellbeing Practitioners (PWPs), from multiple backgrounds including nurse therapists, occupational therapists and social workers. Clinical Psychologists, a Psychodynamic Psychotherapist, a Systemic/Family Therapist and a Veterans’ Mental Health Specialist Practitioner (in collaboration with a charity, Combat Stress) were also engaged in treatment delivery. All therapists complied with codes of conduct set out by their professional or regulatory bodies, such as the Nursing and Midwifery Council. Patients were treated with a range of evidence based interventions including Cognitive Behaviour Therapy (CBT), Eye Movement Desensitization and Reprocessing (EMDR) for dealing with trauma, clinical psychology, psychodynamic psychotherapy; family/systemic therapy; mindfulness and guided self‐help, for example, behavioural activation. The service was able to tolerate higher numbers of cancellations and ‘Did Not Attends’ than is usually tolerated in primary care, in recognition of the higher levels of ambivalence and avoidance many veterans presented with. All veterans retained in the service had to consent to their service records, including medical records, being obtained from the Ministry of Defence. At the end of treatment, or when patients declined further treatment/dropped out, discharge letters were sent to the referrer, General Practitioner and, if requested, the veteran themselves. This paper examines outcomes from the pilot phase of this service through an audit process using routinely generated data.

## 2 The study

### 2.1 Aims

This study aimed to assess whether the MV IAPT service was effective in offering psychological therapies to veterans by comparing symptom levels of depression, anxiety and work and social adjustment at baseline assessment with post‐treatment. The following hypothesis was tested: veterans receiving a full course of designated treatment, as opposed to those still engaged in therapy at evaluation and those who dropped out of the service, would experience a significant reduction in symptoms of depression, anxiety and levels of social maladjustment.

### 2.2 Design

We undertook an observational, prospective cohort study of veterans accessing the MV IAPT service for a pilot period of 20 months from September 2011–April 2013. Data were collected anonymously from the clinical information system of general IAPT services, where standardized measures of depression, anxiety and social adjustment were administered to patients at *each* session and scores entered into the computerized system (Clark, 2011). This enabled measures of severity of each of these domains to be available for most patients even if they dropped out of treatment. After screening for suitability for the service, the outcomes reported here were from patients who were seen at least twice, including an initial assessment, so permitting pre‐ (assessment) and post‐treatment (last available session) scores on the standardized measures to be compared. This definition of treatment, as constituting at least two or more sessions, is in keeping with other reporting of UK IAPT services (Radhakrishnan et al., 2013).

### 2.3 Participants

All veterans referred to the service and receiving an assessment during the period of the evaluation (*n*=707) were eligible for inclusion in the study. However, of these, 505 received more than one appointment. These veterans completed either an agreed period of treatment, had treatment but subsequently dropped out, or were still in treatment at the time data collection ended; these circumstances defined different ‘service conclusion’ groups for analysis. These patients, who had at least two sessions (including an assessment), were therefore likely to have had some form of treatment and so had outcome data available. It is this group that formed the basis of the analysis reported here.

### 2.4 Data collection

Data were collected from symptom questionnaires administered by therapists with results loaded on to the computerized system used for monitoring the progress of therapy. Depression was assessed using the Patient Health Questionnaire (PHQ‐9) (Kroenke, Spitzer, & Williams, 2001). This is a symptom measure so that higher scores indicate more pronounced symptoms, with a range of 0–27. A recommended cut‐off score of 10 or above is indicative of a clinical ‘case’ of depression. Anxiety was assessed using the Generalised Anxiety Disorder scale (GAD‐7) (Spitzer, Kroenke, Williams, & Lowe, 2006). This is also a symptom measure so that higher scores, again, indicate more pronounced symptoms, with a range of 0–21. A score of 8 or above is indicative of a clinical ‘case’ of generalized anxiety, post‐traumatic stress disorder, panic disorder or social anxiety disorder. Social functioning was assessed using the Work and Social Adjustment Scale (WSAS) (Mundt, Marks, Shear, & Greist, 2002). This is a simple 5‐item measure of general impairment drawn from studies of change during psychotherapy. Each question is rated on a scale of 0–8 with higher scores indicating greater impairment, with a score range of 0–40. A total score above 20 indicates moderately severe or worse psychopathology, with scores between 10–20 associated with significant functional impairment. Scores below 10 appear to be characteristic of sub‐clinical populations (Mundt et al., 2002).

### 2.5 Ethical considerations

The study was a service evaluation audit, using routinely collected data and not managed as research in the English National Health Service (NHS); it was therefore not subject to formal ethical review. This was in common with other studies employing IAPT data (Radhakrishnan et al., 2013). However, the study was approved through the local NHS Trust who funded the evaluation to address research governance requirements.

### 2.6 Data analysis

Characteristics of patients receiving the service were first of all compared, in terms of types of service conclusion, using descriptive and inferential statistics. In addition, as an indicator of representativeness, these patient characteristics were compared with those of military personnel more generally, using data from the King's College London military cohort studies (Hotopf et al., 2006), as a test of representativeness.

Outcomes of the service were then assessed by comparing initial assessment with post‐treatment on the above measures for all patients who were accepted into the service (i.e. those receiving assessment and at least one other session). Treatment effect sizes (Cohen, 1988) were calculated by subtracting the post‐treatment score from the pre‐treatment score and dividing by the post‐treatment standard deviation. These provided a standardized, relatively conservative, estimate of impact for the service (Richards & Suckling, 2009) and represent within‐group differences between pre‐ and post‐treatment, the magnitude of which was used as a basis of comparison between different types of service conclusion. Recovery rates (‘% recovered’) were also calculated for those patients initially scoring above the clinical cut‐off points on the above measures who subsequently scored below these cut‐off points for the PHQ‐9 (9 or less) or the GAD‐7 (7 or less).

The study was powered to detect a small to moderate effect on the PHQ‐9 (standardized mean difference=0.3; raw difference of 2 points on the PHQ‐9 at a standard deviation of 7) at alpha=0.05 and 95% power, giving a minimum required sample size of 122 patients. We compared these outcome changes for patients who completed treatment, remained in treatment and who dropped out. Pre‐ and post‐treatment scores were compared using paired *t*‐tests. Categorical variables, comparing proportions of veterans with particular characteristics, were examined using Pearson's Chi square (χ^2^) test or Fisher's exact test. All analyses were conducted using the statistical package for the social sciences (SPSS), version 20.

This study was not a randomised controlled trial and thus potential effects may have been confounded by selection bias and other factors, such as differences in the circumstances of patients. Therefore, these characteristics and outcomes were benchmarked against other studies relating to both interventions and natural recovery rates to draw conclusions about effectiveness.

## 3 Results

**Figure 1:**
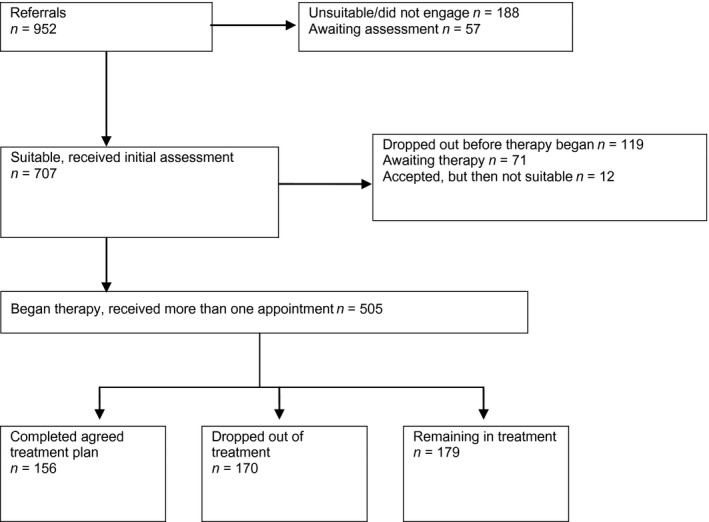
Patient pathways through the clinical service

Figure 1 shows the flow of veterans through the clinical service during the period of this evaluation. Seven hundred and seven patients received an assessment and of those assessed, 505 received more than one appointment. These 505 veterans completed either an agreed period of treatment, had treatment but subsequently dropped out, or were still in treatment at the time data collection ended. These patients, who had at least two sessions (including an assessment), were therefore likely to have had some form of treatment and so had outcome data available. The analysis of clinical outcomes here focuses on this latter group. On average, these patients had nine treatment sessions, with a total of 3,356 hr of contact, including the initial assessment session with a total of 3,243 therapy sessions attended by patients during the evaluation period. For the total sample having received treatment, CBT was the most frequent form of therapy (273 patients) followed by guided self‐help (153 patients), with other treatments being behavioural activation (77 patients), couple therapy (30 patients), interpersonal psychotherapy (28 patients) and counselling (8 patients). These forms of therapy were not mutually exclusive as some patients received more than one type.

### 3.1 Characteristics of patients

Table 1 shows the demographic and other characteristics of patients who received an assessment through the evaluation period. In particular, these patients are split into types of service conclusion arising from the computerized system that collected information pertaining to those going through the service. Thus, sub‐groups of referrals at varying levels of their assessment and treatment are compared against each other. Veterans in each group were, on average, middle‐aged white British males, with a large proportion being married or in a partnership. The majority of patients had served in the Army and only a small proportion from other branches of the forces (RAF and Navy) or reserves. The vast majority had been operationally deployed. Regarding their rank on discharge, most veterans were privates, followed by junior non‐commissioned officers. Veterans who completed treatment did not differ from those who dropped out in terms of their age, gender, ethnicity, marital status; branch of Force, rank on discharge, alcohol misuse and primary referral problem (χ^2^>0.05). However, there were differences in some circumstances between the groups. Those who dropped out of treatment tended to have a forensic history (χ^2^=6.56; *p*<.01), substance misuse (χ^2^=9.56; *p*<.05) and number of unattended sessions (*t*=−3.04; *p*<.01). The most common psychological problems at referral were depression, mixed anxiety and depression and PTSD.

**Table 1:** Characteristics of all patients with two or more sessions

	Completed treatment(*n*=156)	Remaining in treatment(*n*=179)	Dropped out during treatment(*n*=170)
Age, mean (*SD*)	43.6 (12.6)	44.2 (11.1)	41.9 (11.2)
Male	144 (92.3)	170 (95)	153 (90)
White British	147 (96.7)	147 (96.1)	153 (95.6)
Marital status			
Married/civil partnership	64 (41.8)	59 (38.8)	60 (37.5)
Divorced	23 (15)	25 (16.4)	22 (13.8)
Separated	5 (3.3)	10 (6.6)	16 (10)
Single	60 (39.2)	58 (38.2)	61 (38.1)
Branch of force			
Army	129 (83.3)	150 (86.7)	137 (82.5)
Royal navy/marines	13 (8.4)	12 (6.9)	9 (5.4)
Royal air force	9 (5.8)	7 (4)	12 (7.2)
TA/reservists	4 (2.6)	4 (2.3)	6 (3.6)
Rank on discharge			
Private	90 (61.2)	97 (69.3)	98 (66.2)
JNCO	41 (27.9)	23 (16.4)	31 (20.9)
SNCO	10 (6.8)	17 (12.1)	17 (11.5)
Officer	6 (4.1)	3 (2.1)	2 (1.4)
Operationally deployed?	125 (86.2)	113 (88.3)	81 (84.4)
Early service leavers^a^	21 (14.6)	20 (12.4)	16 (10.9)
Forensic history	39 (26.9)	45 (33.8)	54 (41.5)
Has physical disability	23 (16.3)	20 (16.7)	22 (15.3)
Not attended sessions, ‘DNAs’	131 (21)	176 (28)	225 (33)
Misuse			
Substance only	5 (3.2)	13 (7.3)	10 (5.9)
Alcohol only	21 (13.5)	28 (15.6)	16 (9.4)
Substance and alcohol	9 (5.8)	14 (7.8)	21 (12.4)
Primary diagnosis at referral			
Bipolar affective disorder	1 (0.6)	–	4 (2.5)
Depression	48 (30.8)	39 (24.5)	40 (24.9)
Anxiety and Panic	12 (7.7)	10 (6.3)	12 (7.4)
Mixed anxiety and depression	43 (27.6)	46 (28.9)	41 (25.5)
PTSD	43 (27.6)	58 (36.5)	60 (35.3)

Values are *n* (%) unless otherwise stated; Numbers do not always sum up to the total sample size due to missing data; Across the three groups, five veterans were widows.

JNCO, Junior non‐commissioned officer; PTSD, Post‐traumatic stress disorder; SNCO, Senior non‐commissioned officer; TA, Territorial Army.

a Those with four years or less service (Buckman et al., 2013), although recent definitions (Ministry of Defence^,^ 2010) signal that a veteran may be classified as an ‘early service leaver’ if they have served for more than four years but have committed an offence and been discharged from the Services. However, data were unavailable on this aspect and so the former definition was used.

There were several ‘Did Not Attends’ (DNAs) throughout the period of evaluation; overall, there were 619 scheduled sessions with DNAs across the service as a whole with the greatest number of non‐attended sessions (*N*=225) among those who dropped out of treatment and the least (*N*=131) among those who completed treatment. We compared those patients who had at least one unattended session (‘DNAs’) with those who attended every session (‘non‐DNAs’). This was to investigate whether the circumstances of patients prone to non‐attendance might be different. All comparisons, investigating the distribution of characteristics were non‐statistically significant, apart from those with forensic history (χ^2^=3.62, *p*<.05) and those with substance abuse (χ^2^=4.2, *p*<.05) who tended to ‘DNA’; for example, almost 40% of ‘DNAs’ had a forensic history as opposed to 30% of ‘non‐DNAs’.

Comparison of these data, as an indicator of representativeness, with those from the King's College London military cohort studies (Hotopf et al., 2006) shows that those accessing treatment in this clinical service were, on the whole, more likely to be older, single, privates and in the British Army than those in either regular or reserve forces deployed to the Iraq war in 2003; however, proportions of ‘at risk’ groups – reservists, those with physical disabilities and early service leavers – were broadly similar to those of military personnel more generally (Table 2). Comparing veterans who completed treatment with those who dropped out, it was found that those with a forensic history and those with substance misuse tended to drop out of treatment before completion (Fisher's exact test, *p*<.01 for both variables).

**Table 2:** Characteristics of patients accessing treatment in the MV IAPT clinical service compared with UK military personnel in general (King's cohort study)

	Accessing MV IAPT clinical service(*n*=505)	King's cohort study^a^(*n*=4722)
Age		
<25	22 (4)	868 (18)
25–29	60 (12)	994 (21)
30–34	58 (12)	1047 (22)
35–39	51 (10)	896 (20)
40–49	171 (34)	807 (17)
>50	143 (28)	110 (2)
Male	467 (92)	4344 (92)
Marital status		
Married/civil partnership	183 (36)	3560 (75)
Divorced/previously married	70 (13)	277 (6)
Single	179 (35)	864 (19)
Branch of force		
Army	416 (82)	3066 (64)
Royal navy/marines	34 (7)	761 (17)
Royal air force	28 (6)	895 (17)
Reservists	14 (3)	786 (9)
Rank on discharge		
Non‐commissioned officer	139 (27)	2962 (63)
Commissioned officer	11 (2)	814 (17)
Private/other rank	285 (56)	904 (20)
Early Service Leavers	57 (11.2)	80 (9.5)^b^
Has physical disability	65 (12.8)	575 (12)

Values are *n* (%); Numbers do not always sum up to the total sample size due to missing data.

a From sample engaged in Operation TELIC 1, representing the build‐up and completion of operations in the 2003 Iraq war (Hotopf et al., 2006).

b From *n*=845 sample investigated by Buckman et al. (2013).

### 3.2 Patient outcomes

Data were available on pre‐ (assessment) and post‐treatment (last available session) standardized measures for the 505 veteran patients accessing the service and receiving some form of treatment. Table 3 shows the scores on standardized measures, effect sizes and recovery rates for depression and anxiety for those who completed treatment as agreed, those remaining in treatment and those that dropped out prematurely. Overall, across the whole sample, there were highly significant improvements on all measures: PHQ‐9 (*t*=12.84, *df* 339, *p*<.001), GAD‐7 (*t*=12.53, *df* 339, *p*<.001) and WSAS (*t*=7.89, *df* 333, *p*<.001). Pre to post effect sizes were moderate to large: 0.62 for the PHQ‐9; 0.63 for the GAD‐7; 0.41 for the WSAS. These figures compare favourably with, albeit slightly lower than, the effect sizes for general IAPT services, where effect sizes of 1.26 for the PHQ‐9 and 1.25 for the GAD‐7 have been reported (Clark et al., 2009). An overall impact, in terms of effect size, is also greater than that from trials of anti‐depressants, where an effect size of 0.42 has been reported from a meta‐analysis (Arroll et al., 2005).

**Table 3:** Outcomes for sub‐groups of patients (‘service conclusion’) assessed in the clinical service

	Completed treatment(*n*=156)	Remaining in treatment(*n*=179)	Dropped out during treatment(*n*=170)
Pre PHQ‐9, mean (*SD*)	15.3 (6.7)	17.9 (6.1)	16.9 (6.4)
Post PHQ‐9, mean (*SD*)	7.7 (7.1)	14.8 (7.4)	14.6 (7.2)
Effect size	1.07	0.42	0.32
Pre GAD‐7, mean (*SD*)	13.3 (5.8)	15.1 (5.1)	14.6 (5.3)
Post GAD‐7, mean (*SD*)	6.7 (6.4)	12.2 (5.9)	12.9 (6.1)
Effect size	1.03	0.49	0.28
Pre WSAS, mean (*SD*)	18.7 (10.8)	22.8 (10.7)	21.3 (10.6)
Post WSAS, mean (*SD*)	10.7 (10.7)	20.3 (11.7)	19.7 (10.7)
Effect size	0.75	0.21	0.15
Pre rate of depression, *n* (%)	116 (84)	98 (88)	80 (88)
Post rate of depression, *n* (%)	50 (36)	81 (73)	66 (72)
Recovery rate, depression %	57	17	17
Pre rate of anxiety, *n* (%)	116 (84)	99 (89)	79 (87)
Post rate of anxiety, *n* (%)	53 (38)	83 (75)	70 (77)
Recovery rate, anxiety %	54	16	11

Proportions are valid percentages reflecting only those with both pre‐ and post‐treatment data available. The sample sizes were therefore: Completed treatment, *n*=138; remaining in treatment, *n*=111; dropped out, *n*=91.

Effect size is standardized within‐group difference between pre‐ and post‐treatment; the magnitude of which is the basis of comparison between difference service conclusion groups.

In terms of recovery, overall 294 of the 340 patients for whom we had both pre‐ and post‐treatment data could be classified as clinical cases of depression on the PHQ‐9 at initial assessment. Of these, 97 (33%) had recovered (i.e. were now scoring lower than the clinical cut‐off score). The figures for anxiety overall were 294 of the 340 patients classified as clinical cases on the GAD‐7 at assessment with 88 (30%) having recovered.

For the different types of service conclusion, there were highly significant improvements on all measures: PHQ‐9, GAD‐7 and WSAS (at *p*<.01). Pre to post effect sizes were in a hierarchy, from the highest for those who completed treatment, to those remaining in treatment, to drop outs with the lowest effect sizes. Recovery rates mirrored this pattern. Those having completed treatment had a higher recovery rate from depression (57%) than those remaining (17%) and those who dropped out (17%). The recovery rates for anxiety were: for those having completed treatment (54%), those remaining (16%) and those who dropped out (11%). These recovery rates compare favourably with those of general IAPT services from evaluations in demonstration sites (Clark et al., 2009), where rates of 52% were reported. They also comfortably exceed the 5–20% reported for natural recovery or minimal intervention for cases with a prior duration of illness of 6 months or over (Clark et al., 2006; Posternak & Miller, 2001).

## 4 Discussion

This study has presented several key findings related to outcomes for patients of a veteran‐specific mental health service, employing the general framework of the IAPT model in the UK. The veterans accessing the service were, in several important respects relating to risk of psychological disorder (i.e. reservists, those with physical disabilities and early service leavers), similar to those from large‐scale surveys of the veteran population. However, the patients treated in this service tended to be older, single veterans of lower rank than those surveyed as accessing standard primary care services. The impact of this dedicated, specialist psychological service was good; beneficial patient outcomes were comparable to those reported from general IAPT services and exceeded the natural recovery rates for depression seen in waiting list control groups and those for anxiety from randomised trials of CBT (Clark et al., 2006; Posternak & Miller, 2001). The therapies undertaken therefore appeared to bring about benefits for the patients. There was also a significant ‘dosage’ effect; those who completed a full course of therapy did better than those who dropped out and those who were still engaged in treatment during the evaluation period.

The service was tailored to the specific needs and presentations of military veterans and, as a consequence, tended to allow access to groups not traditionally served by general IAPT services, such as those with substance abuse. The service also tolerated cancellations of appointments and ‘Did Not Attends’; for example, of the scheduled sessions with DNAs across the service as a whole, there were a large number, 131, from those who subsequently completed treatment. This suggests that the clinical service ‘holds on’ to patients and remains engaged with them until therapy completion, even if the patient did not arrive for specific sessions during treatment. In this respect, the service appeared to target interventions towards those veterans at greater risk of mental health problems such as early service leavers. However, those with a forensic history or substance misuse tended to drop out of the service early. Thus, although the service endeavoured to engage these hard to reach groups, there was a need for specific responses to them, such as more assertive engagement, as veterans are often reticent to seek help or present particular challenges to services (Graham & Livingston, 2011; Iversen et al., 2010).

These findings have implications for nurse therapists and others, working with this vulnerable patient group. In contrast to countries like the United States, where a dedicated infrastructure exists for veterans’ healthcare needs, the lack of specifically tailored help in the UK has been problematic. Many veterans have been confused by the different services on offer, their acceptance criteria and referral routes (Macmanus & Wessely, 2013). One response has been to enshrine access to appropriate health treatment for veterans in law (Ministry of Defence 2011); however, appropriate services have been slow to develop. The IAPT approach to providing psychological therapies, configured specifically towards the needs of veterans, may offer much in this context of increased commitment to the needs of the veteran population. Specialist nurse therapists, in particular, have embraced the approach (National Institute for Mental Health in England 2010). However, specialist nurses will have to modify their treatment approaches somewhat as this population offer particular challenges, in particular nurses will have to be aware of and make efforts to counter resistance from this population, who are used to seeing themselves as ‘fit and well’ and may not engage with therapy quickly. Veterans are often reticent to seek help and may have a forensic history along with drug and alcohol misuse, which means they may be particularly ‘hard to reach’. Nurses would have to draw on examples of psychological approaches that endeavour to engage with such particularly vulnerable and perhaps treatment resistant patients. Strategies such as maintaining a ‘holding environment’, setting limits and providing structure for the patient are suggested by interventions internationally (Koekkoek, van Meijel, & Hutschemaekers, 2006). Specialist nurse therapists may need support in transferring their skills to this patient group, through guidelines, clinical supervision and audit by senior nurse managers (Butler, Begley, Parahoo, & Finn, 2014).

### 4.1 Limitations

There were, of course, limitations to this study. As a routine evaluation from veterans’ continued use of the service it was not a controlled study and in particular, did not employ a control group of patients who were not receiving the service. It must, however, be noted that this is a real limitation of many evaluations of psychological therapies that have taken place, such as those of general IAPT services (Clark et al., 2009). For the veteran samples studied here, scores at assessment on the standardized measures were in the high range of scores found in other evaluations and the possibility of regression to the mean (Nesselroade, Stigler, & Baltes, 1980), whereby very vulnerable and/or ill participants have only one direction of travel – that of improvement – cannot be discounted. However, against this, the rates of improvement in the outcome indicators employed here are well above the rates for natural recovery or ‘spontaneous remission’ shown in other studies, for example, remission rates of 23% of cases of untreated depression within 3 months and 32% within 6 months (Whiteford et al., 2013), as against 57% for those who completed treatment in the present study.

## 5 Conclusion

The findings reported here have much to offer in the context of providing salient evidence, in particular to those commissioning mental health services, against the backcloth of an increased commitment to the veteran population, such as that in the Military Covenant, now enshrined in law (Forster, 2012). Commissioners will need to balance competing priorities for funding against this overarching commitment to the specific needs of veterans as they access health services. The evidence of outcomes offered here, from one model of service delivery addressing the mental health needs of veterans, may assist in this process providing data on a significant number of individuals’ responses to a veteran‐specific intervention. This may be particularly helpful when these outcomes are set against the additional costs involved, mostly from employing therapists with knowledge or training around the specific needs of veterans.

In general, it would appear that the MV IAPT service appears to offer an effective intervention to veterans presenting with psychological disorder. Given the recent widespread commitment to developing mental health services that target the specific needs of veterans, such an approach is one model for which, from this pilot evaluation, evidence exists of beneficial outcomes in terms of health and social functioning.

## Funding

This work was supported by a grant to PSSRU, University of Manchester from Pennine Care NHS Foundation Trust.

## Conflict of interest

PC, CG and DC: No conflicts of interest have been declared by the authors. PD, AB and HL are employed by Pennine Care NHS Foundation Trust; AB and HL work in the clinical psychological therapies IAPT service for military veterans.

## Author contribution

All authors have agreed on the final version and meet at least one of the following criteria [recommended by the ICMJE (http://www.icmje.org/recommendations/)]:

- substantial contributions to conception and design, acquisition of data, or analysis and interpretation of data;
- drafting the article or revising it critically for important intellectual content.
